# Optimising symptom management in children with cancer using a novel mobile phone application: protocol for a controlled hybrid effectiveness implementation trial (RESPONSE)

**DOI:** 10.1186/s12913-021-06943-x

**Published:** 2021-09-09

**Authors:** Natalie Bradford, Paula Condon, Erin Pitt, Zephanie Tyack, Kimberly Alexander

**Affiliations:** 1 Cancer and Palliative Care Outcomes Centre, School of Nursing, Queensland University of Technology, 60 Musk Avenue, Kelvin Grove, QLD 4059 Brisbane, Australia; 2 Centre for Children’s Health Research, Children’s Health Queensland Hospital and Health Service, Queensland Children’s Hospital, 62 Graham St, South Brisbane, QLD 4101 Brisbane, Australia; 3 Centre for Healthcare Transformation, Faculty of Health, Queensland University of Technology, 60 Musk Avenue, Kelvin Grove, QLD 4059 Brisbane, Australia

**Keywords:** Child, Adolescent, Cancer, Patient Reported Outcome, Digital health, Mobile health, Symptoms and side effect, Health services research, Implementation, Trial

## Abstract

**Background:**

Intense and aggressive treatment regimens for most children’s cancer have achieved vast improvements in survival but are also responsible for both a high number and burden of symptoms. The use of Patient Reported Outcome Measures (PROMs) demonstrates a range of benefits for improved symptom management in adults with cancer. There are, however, multiple barriers to integrating PROMs into routine care in children and adolescents with cancer. This study aims to evaluate: (1) the effectiveness of electronic PROMs to generate stratified alerts, symptom management recommendations and graphical summaries (the RESPONSE system) to improve health outcomes and (2) the implementation of the RESPONSE system by assessing feasibility, acceptability, satisfaction, and sustainability.

**Methods:**

A pragmatic hybrid II effectiveness-implementation controlled trial, using mixed methods, will be undertaken, advancing both knowledge of the effectiveness of the intervention and implementation factors. One-hundred and sixty children with cancer receiving active treatment will be recruited 1:1 to a non-randomised study involving two groups with an equal number of participants in each group. The intervention group (*n* = 80) will be prospectively recruited to receive the RESPONSE system intervention over eight weeks, versus the historical matched control group (*n* = 80) who will complete the ePROMs without access to the RESPONSE system. The primary outcome of the effectiveness trial is change between groups in total symptom burden. Secondary outcomes include child health-related quality-of-life and implementation outcomes. Trial data will be analysed using linear mixed-effects models. Formative implementation evaluation is informed by CFIR and ERIC frameworks and implementation outcomes will be mapped to the RE-AIM framework and include interviews, field notes, as well as administrative data to evaluate feasibility, acceptability, satisfaction and sustainability.

**Trial registration number:**

ACTRN12621001084875. Retrospectively Registered 16 August 2021.

## Background

Children undergoing treatment for cancer experience multiple, persistent and distressing symptoms related to cancer and treatment [1, 2]. Up to 30 % experience ten or more symptoms that persist up to two weeks following chemotherapy [3]. However, children and their caregivers may perceive this high symptom burden to be inevitable or even a sign that treatment is working, and thus may not report symptoms amenable to intervention [4]. Further to this, symptoms are also often undetected and undertreated by clinicians [5]. As more treatment is delivered in the ambulatory care setting, children often suffer symptoms at home, away from clinical care [6], resulting in perceptions that there are few options for intervention [7]. This limits opportunities for patients and caregivers to receive support and education from clinicians to manage symptoms effectively. Caregivers are expected to take on the responsibility of managing their child’s symptoms, yet there are few resources to support this, and they often report feeling left alone [8].

It is evident, that symptom management is sub-optimal, causing both unnecessary distress and avoidable hospitalisations [9]. Poor symptom control affects treatment tolerance and can cascade into longer-term problems that negatively impact both the child and their families’ quality-of-life [10–12]. Furthermore, poor symptom control is associated with poorer psychological outcomes [13] and PTSD that may not emerge for years following treatment [14]. New ways are needed to support children and their caregivers to communicate with healthcare providers and receive management recommendations about distressing symptoms, regardless of the setting in which care is provided.

The use of Patient Reported Outcome Measures (PROMs) in routine clinical care can overcome barriers by normalising symptom reporting processes, placing value on patient experience and generating timely, reliable data on which to act [4]. PROM responses about health come directly from the patient, their proxy, or in the case of children co-completion with the caregiver is advocated [15]. There is compelling evidence for the effectiveness of PROMs to improve quality of care, shared decision making, and communication at the individual level [16–18]. Additionally PROMs can improve health at the population level through reduced costs, and enhanced clinician satisfaction with work [19].

There are, however, multiple barriers to integrating PROMs into routine care for children at the individual, clinical, organisational and systems level [4]. At the individual level, the child’s developmental stage, their understanding of symptoms, and the availability of child-friendly measures present barriers. At the clinical level, outcomes that matter to healthcare providers (symptoms that delay treatment) may not be the same as those that matter to children (concerns with body image and feeling anxious) [2, 5]. Clinicians may also be concerned about resources, and the burden versus benefit value proposition [20]. From an organisational perspective, technological barriers prevent the integration of electronic data from PROMs (ePROMs) into clinical workflow processes, management plans and medical records, limiting feasibility and acceptability [16]. From a systems perspective, there is questionable financial incentive to integrate PROMs as well as uncertainty about implementation approaches; given the expense and logistical challenges, health services are understandably cautious [21].

These barriers and other implementation factors can be examined using tools from implementation science. Multiple frameworks guide the implementation evaluation of the intervention, which includes a systematic, formative and summative evaluation that considers the context, causal assumptions and mechanisms of impact. The elements and constructs from implementation frameworks are useful for mapping barriers to implementation, developing strategies to mitigate identified barries and also to evaluate the implementation outcomes; understanding all these phenomena can ultimately facilitate the sustainability and scalability of interventions [22, 23].

Accordingly, we report the protocol of a study to evaluate the effectiveness and implementation outcomes of an ePROM intervention targeting symptoms in children undergoing systemic cancer treatment – the RESPONSE system. The intervention involves multiple components including: (1) weekly monitoring of symptoms using a validated, child friendly ePROM (SSPedi) [24, 25]; (2) stratified alerts for symptoms that reach pre-determined thresholds requiring intervention; (3) evidence-based symptom management recommendations, and (4) graphical displays of SSPedi information enabling symptom trends to be visualised over time. The comparison intervention involves completion of ePROMs alone, without stratified alerts, symptom management recommendation or graphical displays.

## Methods and Design

### Aims and objectives

The primary aim is to determine the effectiveness of the RESPONSE system intervention on the total symptom burden of children receiving cancer treatment. Secondary aims are to assess effects on health-related quality-of -life and implementation outcomes.

### Hypothesis (effectiveness component)

The implementation of ePROMs, stratified alerts, provision of symptom management recommendations, and graphical displays using the RESPONSE system will reduce the total symptom burden experienced by children receiving treatment for childhood cancer compared to the ePROM alone intervention.

### Implementation outcomes

Implementation outcomes include the feasibility, acceptability, satisfaction and sustainability of implementing the RESPONSE system intervention. The contextual factors and core components of the intervention will be identified for inclusion in a future large-scale randomised controlled trial.

### Context and setting

The setting for the study is a children’s cancer centre at a major metropolitan hospital in Australia. The centre receives approximately 200 children newly diagnosed with cancer each year, and treatment can take up to three years or longer to complete. The catchment of the hospital includes rural, regional and metropolitan areas and has formal networks established with 12 smaller regional hospitals that provide supportive children’s cancer care. Caregivers (and their eligible child with cancer) will be consecutively approached and recruited to the study.

### Development of the intervention

The RESPONSE system was iteratively developed with clinicians, children and caregivers throughout 2019–2020. Firstly, a clinical reference group was established comprising 12 oncology medical, nursing and allied health staff who met at least monthly through intervention development. The first objective was to review available PROMs described in systematic reviews [6, 26, 27]. The SSPedi was chosen because of its ease of use with children, brevity and due to its intended use as a routine measure for symptom assessment, rather than as a proxy measure or end-point for other intervention studies [22, 24, 25]. SSPedi uses a 5-point Likert scale to measure the degree of bother either today or yesterday (0 = not at all − 4 = extremely bothered) for 15 different symptoms. The measure is validated for children 8–18 years, their caregiver proxy, and for ‘co-completion’ by both child (4–18 years) and caregiver together [15]. Cognitive interviews were completed with children and caregivers to confirm the content validity of SSPedi and its relevance to the Australian population [25].

### Stratified alerts, symptom management recommendations and graphical trends

The clinical reference group iteratively developed algorithms to determine thresholds for each of the 15 symptoms measured by SSPedi. Each symptom was individually appraised for the level of potential risk, with each possible answer of bother determining one of three responses regarding management. The algorithms include reference to previous SSPedi completion within the previous 14 days, as well as diagnosis. The final algorithms were ratified by oncologists, nursing and allied health staff at the children’s cancer centre.

Evidence-based symptom self-management recommendations were drafted from clinical practice guidelines (where available) by the clinical reference group and endorsed by medical, nursing and allied health oncology clinicians working in the study setting [28]. Recommendations were appraised for ease of reading, ensuring content was at an appropriate reading level (grade 8) or below. Wording was also reviewed by two adolescents (age 13 and 15 years) for content and language resulting in some changes. A consensus was obtained through discussion with the clinical reference group to graph SSPedi both for one-time completion (visualising all symptoms on a bar graph), as well as an option to select up to three symptoms to view over a 1-week, 4-week and 12-week time period. These time periods were chosen to correspond with common chemotherapy protocols in children’s cancer. All components were then incorporated into the design of a mobile application (app).

### Mobile Application Design

**Fig. 1:**
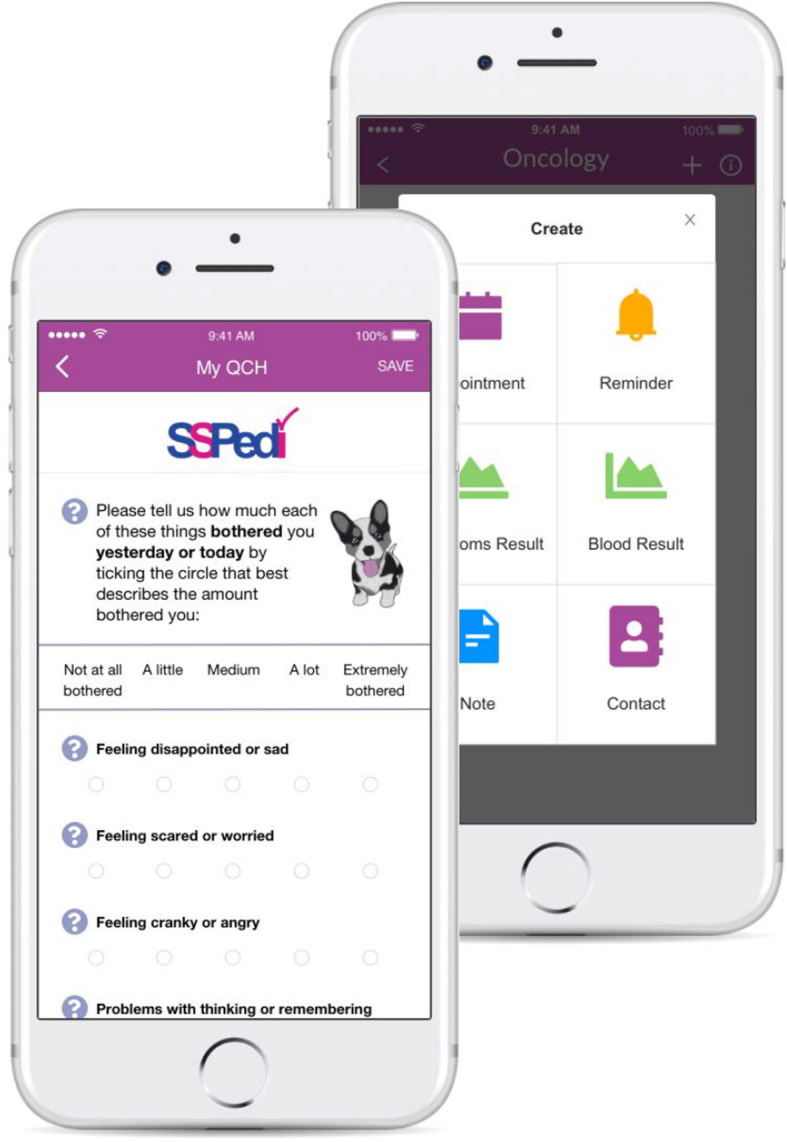
Screen shots of the RESPONSE mobile phone app

Along with digital technology experts and input from caregivers of children with cancer, a subgroup of the clinical reference group (n = 5) iteratively conceptualised and developed the design for the app. Wireframe screens were developed using online software, and these were presented to stakeholders, with feedback used to make modifications. Cognitive interviews were undertaken with nine caregivers and two adolescents with cancer using ‘think-aloud’ techniques in order to test the design of the app [29]. Changes to the design were made based upon feedback, such as including the use of more colour, wording and adding places for caregivers to record notes (Fig. 1).

After caregivers register within the app and view the introductory welcome screens, the ePROM is completed by the participant by selecting the appropriate response corresponding to the level of bother for each symptom. After completion, the algorithm determines the stratified alert using a traffic light system: green - no concern; amber - concern that requires discussion with the clinical team at the next clinic visit, and red - concern that requires immediate discussion with the clinical team, with caregivers prompted to phone the hospital. Relevant symptom management recommendations are then presented within the app. Graphical displays of all symptoms can be viewed at any time.

Once the design of the app was completed, it was provided to the Information Communication Technology partner of the Children’s Hospital for development. The backend of the app database is stored on an approved secure and private cloud server within Australia, that meets the requirements for the protection of participants in relation to confidential processing, collection, and access to personal data. A web-based portal provides access for clinical staff to manage users, facilitate push notifications (for example, reminders to complete ePROMs) and tracking in a convenient format. While these data are available to clinicians, they are not linked to medical records and the RESPONSE system is designed as a caregiver and child patient-facing app, providing information directly to the user.

### Pre-implementation planning

Interviews were conducted with clinicians as part of the pre-implementation planning. Interview questions were informed by the Consolidated Framework for Implementation Research (CFIR) [30] (Table 1). Findings are used to iteratively develop, design and guide strategies for implementation using the ERIC Expert Recommendation for Implementing Change (ERIC) [31] compilation of strategies. The CFIR-ERIC Implementation Strategy Matching Tool was used to facilitate this process [23]. For example, we identified a potential barrier of nursing staff not having access to relevant information regarding evidence-based symptom management for all the symptoms screened with SSPedi. Accordingly, education modules were developed to support nursing symptom management education at novice, intermediate and advanced levels. To ensure organisational support, presentations of the project were made to oncology medical and nursing and allied health staff, hospital executives, the state-wide clinical networks, and the family centred care committee (Table 2).

**Table 1:** Pre-implementation formative evaluation clinician interview questions

Interview questions to clinical staff	CFIR Domain
What is your understanding of the RESPONSE intervention to support symptom management in children with cancer?	CFIR/Characteristics of Individuals/Knowledge and Beliefs about the intervention
What is your understanding of how the intervention will be integrated into current practices?	CFIR/Inner Setting/Implementation Climate/Compatibility
Do you feel that you have the current knowledge level and skills to answer potential calls from caregivers regarding symptoms identified by the SSPedi Tool?	CFIR/Inner Setting/Readiness for Implementation/Access to knowledge and information
Do you think educating patient and /or caregivers in how to access and use the RESPONSE intervention is part of your role?	CFIR/Inner Setting/Implementation Climate/Relative Priority
Are there other initiatives in your workplace that have higher priority?	CFIR/Inner Setting/Implementation Climate/Relative Priority

*CFIR* Consolidated Framework for Implementation Research

**Table 2:** CFIR-ERIC Implementation Strategy [23] examples

*CFIR Potential Barriers Identified*	*ERIC Strategies*
Intervention characteristics: • Stakeholders perception of RESPONSE intervention unclear • Stakeholders believe the innovation is complex based upon disruptiveness and number of steps needed to implement	• Assess for readiness an identify barriers and facilitators • Create a learning collaborative, tailor strategies, promote adaptability
Inner setting • Cultural norms, values and assumption may hinder implementation • There is little motivation for change, and no expectation that use of the innovation will be rewarded, supported or expected	• Involve Executive Boards • Inform local opinion leaders • Identify early adopters • Conduct local needs assessment • Designate and train for leadership
Process • Little or no quantitative and qualitative feedback about the progress and quality of implementation nor regular debriefing about progress and experience	• Audit and provide feedback • Develop and implement tools for quality monitoring • Develop formal implementation blueprint

*CFIR* Consolidated Framework for Implementation Research [30]

*ERIC* Expert Recommendation for Implementing Change compilation [31]

### Study design

A pragmatic hybrid II effectiveness-implementation, controlled trial and implementation study was designed to evaluate the RESPONSE system intervention. A hybrid design advances knowledge of both the effectiveness of the intervention and the implementation factors associated with integration into clinical practice [32]. This is the best available method to collect high-quality comparative data, and expedite findings and translation into clinical practice. For ethical reasons, a non-randomised design was chosen, as the children’s cancer centre endeavoured to make the app available to all children and families. Hence, a matched control group is being recruited during the development of the app; non-randomised controlled trials are commonly used in children’s cancer studies [33, 34]. An embedded process evaluation mapped to the RE-AIM framework [35] is included to assess implementation outcomes including acceptability, fidelity and satisfaction (Table 3). This involves interviews with caregiver and child participants as well as clinicians. It also involves collecting fields notes, and relevant clinical and administrative data.

**Table 3:** Implementation outcome measures mapped to the RE-AIM framework [44]

Setting	RE-AIM domain	Data collected	Timing
*Primary outcome*:			
	**EFFECTIVENESS**		
Child/Caregiver	Total symptom burden	SSPedi	Weekly for 8 weeks
*Secondary outcomes*			
	**EFFECTIVENESS**		
Child/Caregiver	Health-related quality-of-life	PedsQL Cancer Module	Baseline, week 4, week 8
Child/Caregiver	Distress thermometer	Distress Thermometer	Baseline, week 4, week 8
QCH records	Economics- health service utilisation	Unplanned hospital admissions	Audit at completion
Child/Caregiver	Experience of care, acceptability, satisfaction	Semi-structured interviews	Completion of recruitment
	**REACH**		
QCH	Percentage uptake of RESPONSE system among eligible children	Log data from recruitment database	Audit at completion of recruitment
Medical record	Percentage of eligible children recruited Clinical and demographic characteristics	Log data from recruitment database	At recruitment
Service providers	Proportion of staff reached by educational presentations	Structured interview	At completion of data collection
	**ADOPTION AND MAINTENANCE**		
Child/Caregiver and Health care providers	% eligible children/caregivers/ healthcare providers educated to use RESPONSE system	Log data from intervention database	Audit at completion of recruitment
Service providers	% aware of RESPONSE system% use RESPONSE data in clinical consultations	Log data from intervention database	Audit at completion of data collection
Child/Caregiver	Fidelity to protocol	Data from intervention database	Audit at completion of data collection
Child/Caregiver	Number of links to management recommendation opened	Dashboard for app	Audit of app data at completion
Service providersChild medical records	Number of alert generated contacts receivedNumber of interventions/ referrals made	Dashboard for appQCH records	Audit at completion of data collection
	**IMPLEMENTATION**		
Child/Caregiver	Acceptability, satisfaction	Structured interview	At completion of data collection
Service providers	Acceptability, satisfaction	Structured interview	At completion of data collection
QCH	Documentation of symptom burden and management	QCH records	Audit completion of data collection
QCH	Number of hospital presentations/clinical encounters	QCH records	At completion of data collection

*QCH* Queensland Children’s Hospital; *RE-AIM *[44] Reach, Effectiveness, Adoption, Implementation Maintenance

### Participants

Participants will be consecutively recruited from the Children’s Cancer Centre Day Oncology Unit by research nurses not involved in the clinical care of children. We have previously described in detail our recruitment strategies with this population group [36]. Recruitment to the control group commenced in January 2020.

### Inclusion criteria

Children aged 4 to 18 years, receiving active treatment for blood cancer or solid tumors and their caregiver/s are included. Active treatment for cancer is defined as receiving either planned cycles of chemotherapy or radiotherapy. Participants will be recruited within two weeks of a planned treatment cycle. Prior to recruitment, their clinical status and the appropriateness of approaching potential participants will be confirmed with the treating clinicians. Participants are required to be able to read and understand English, and to have access to a smartphone, iPad or computer and Internet.

### Exclusion criteria

Children with brain cancer are excluded from this study due to multiple competing studies currently being undertaken in children’s brain cancer at the cancer centre and the concern of overburdening participants. Children who are receiving Hematopoietic Stem Cell Transplantation are also excluded from the current trial due to the complexity of managing their disease and side effects. Children with advanced disease, or disease progression are excluded from the current trial. Furthermore, participants will be excluded if there is difficulty with obtaining consent (for example, the child is under child protection orders), or if there are difficulties understanding English. While inclusion of versions for non-English speaking families is ultimately our goal, this first version is developed using tools validated in the English language.

### Sample size

The sample size is based upon feasibility. Considering previous research [37], we anticipate 50 % of participants will meet the eligibility criteria (many children diagnosed with cancer are aged under 4 years) and that 80 % will consent to the study, meaning over a 12-month time period, approximately 80 participants per group can be recruited. To account for 20 % attrition, recruitment will continue until there are 96 participants in each group. In terms of clinical effectiveness, a difference of 2.7 on the SSPedi scale is considered clinically meaningful at the individual level [24]. Data from this current study will be used to calculate a sample size powered to detect clinically meaningful differences in a larger future study.

### Control Group

The control cohort recruited during the development of the app receives usual care with no access to the RESPONSE system. Data collection for the control group commenced in January 2020 but was interrupted and paused because of COVID-19 for several months and is ongoing; recruitment will continue until the app is released and tested prior to commencing the trial.

### Intervention group

Intervention participants will receive usual care, supplemented by symptom monitoring through completion of SSPedi, stratified alerts based upon the pre-defined algorithms, access to symptom management recommendations and graphical displays of symptom trends over time through the RESPONSE system (Fig. 1). Recruitment will continue until 1:1 matching is achieved on disease type of the recruited controls.

### Procedures

All participants complete the SSPedi ePROM at the start of a treatment cycle weekly for eight weeks, along with other validated measures. Control group participants complete all measures, including SSPedi through REDCap™ and receive up to three reminders sent via email or text message. Intervention participants complete SSPedi using the mobile RESPONSE app, reminders pop-up notifications occur at 24 and 48 h if SSPedi is missed. The dashboard is used to schedule SSpedi timing and the reminder notifications. Other validated measures in the intervention group are collected via REDCap™ [38]..

### Effectiveness outcomes and evaluation

The primary outcome is total symptom burden measured by weekly completion of SSPedi by the child participant, caregiver proxy or child and caregiver co-completion. Secondary outcomes include health-related quality-of-life (PedsQL Cancer Module) [39], the Pediatric Distress Thermometer [40], and FACES pain scale [41]; these measures are collected at baseline, weeks four and eight post-baseline again by the child participant, caregiver proxy or child and caregiver co-completion. The PedsQL Cancer Module is a validated tool with 32 items for cancer specific health related Quality of Life measurement in children and adolescents aged 2–18 years (child and caregiver proxy) [39]. The Pediatric Distress Thermometer is a one item screening tool that measures distress, defined as worry, anxiety, sadness, or fear, reported by the child on a visual analogue scale from 0 (no distress) to 10 (severe distress), based on the child’s experience in the previous week [40]. A recent study has identified threshold scores for children with cancer [42]. The FACES pain scale also uses a visual analogue scale to assess intensity of pain and is validated from age 4 years onwards (and caregiver proxy). The FACES pain scale conforms to a linear interval scale from 1 to 10 scale [41].

Data collected regarding the child includes gender, age at diagnosis, age at study enrolment, diagnosis, treatment, and complications during the study period; all of which will be collected from medical records.

### Effectiveness analysis

Changes in the primary outcome of total symptom burden between the intervention and control group will be descriptively reported and examined using mixed linear mixed-effects models accounting for repeated observations from the same child. Secondary outcomes of quality of life, distress and pain will also be examined with mixed effects regression. This will allow for exploration of effects both within and between individuals, as well as the intervention and control groups. Potentially confounding co-variables will be included, particularly if any demographic or clinical differences are identified between groups at baseline. Outcomes will be stratified by the person completing the measures (caregiver, child participant or co-completion) and gender to determine whether effects differ based upon these factors. A sensitivity analysis will also be conducted, where possible, to compare results between caregiver versus child participant report [43]. The amount and types of missing data will be descriptively reported. Missing data at random will be imputed, and a sensitivity analysis will be completed to investigate possible sources of bias due to missing data. The characteristics of symptom burden over the eight-week study duration, as well as any pharmacological or non-pharmacological interventions or referrals provided to address symptoms will be described. Quantitative data analysis will be undertaken with IMB SPSS version 26.0, Released 2019, Armonk, NY:

### Implementation outcomes and evaluation

Outcome measures for elements of RE-AIM/PRISM [44] includes data from the RESPONSE app, stakeholder and participant interviews and health service administrative data. To provide preliminary data regarding the cost effectiveness of the intervention an economic evaluation will compare healthcare utilisation between groups, and fixed and variable costs of the intervention to the health service.

Implementation evaluation of the RESPONSE app will be determined by data including the behaviour of users (frequency of app use, completion of SSPedi, activation of alerts). Participants will be asked to keep a log diary of any technical difficulties encountered. The feasibility, acceptability and safety of the RESPONSE app will be explored in semi-structured interviews with a subset of recruited child participants, caregivers and clinicians. Fidelity of the intervention will be assessed through study records.

### Implementation Analysis

Review of logged data will provide information regarding the ability of the RESPONSE system to capture symptom burden, completion rates of the SSPedi tool, generation of stratified alerts, access of symptom management recommendations and review of graphical displays. Each event on the app will be collected, so that the total count of events can be calculated over the study time frame. Data analysis will be undertaken per participant as well as across the whole study and across a specific time frame (e.g. one day). Reliability of the RESPONSE system to provide appropriate care management recommendations based on symptom scores will be evaluated with test-retest reliability (intraclass correlation coefficients for repeated measures within the same person) between consecutive days.

Process evaluation will consider causal pathways, safety aspects and mechanisms influencing implementation into clinical practice. Experiences of the intervention from both participants’ and clinicians’ perspectives will assess feasibility, appropriateness, acceptability, and satisfaction with the RESPONSE system for symptom management. Qualitative data will be analysed using interpretive description, an inductive analytical approach to understand clinical phenomena that yield applications implications [45]. Finally, Framework analysis [46] will be used for quantitative and qualitative data. These data will be mapped to the constructs of RE-AIM as overarching themes in matrices, where columns are codes and rows are participants [46]. Positive and negative quotes and descriptive data will be examined for each construct in the RE-AIM framework to determine influences on implementation. NVivo (released in March 2020) [47] will be used to manage qualitative data analysis. Successful implementation is defined a priori as:

- > 50 % of intervention participants completing 80 % of scheduled SSPedi measures.
- > 50 % of intervention participants acting appropriately upon alerts to contact clinical teams.
- > 50 % of intervention participants accessing relevant symptom management advice.
- > 50 % of intervention participants discussing RESPONSE data in clinical consultations..

### Management of participant data

Participants will be provided with written information sheets and have the study verbally explained to them, including the identified risks, benefits, confidentiality and process for consent. All data collected from participants will be coded, and identifying information will be removed. Data will be stored on password-protected files accessible only by the research team.

### Ethical considerations

Ethical approval for this study was obtained from the local Children’s Health Queensland Hospital and Health Service Human Research Ethics Committee (Ref HREC/18QRCH/18). Written informed consent is obtained from the parent or caregiver for any participant under 18 years old. Any participants who choose not to participate or withdraw after providing consent will be assured their decisions will not affect their relationship with their healthcare providers or the hospital. Data will be stored, archived and destroyed in accordance with the National Health and Medical Research Council’s Australian Code for the Responsible Conduct of Research. No identifiable data will be included in the dissemination of results.

### Dissemination

Research outcomes will be disseminated formally through high-ranking peer review publications and conference proceedings. Outcomes will also be presented at the Oncology Family Forum meetings and shared at hospital education meetings. A summary of the final combined results will be available to participants.

## Discussion

Harnessing technology to screen, measure and report ePROMs in real time is changing clinical practice around the world [6, 48, 21]. Electronic systems provide new ways for patients to report symptoms outside clinic visits [49], reduce errors in reporting and are efficient for managing data [50]. This study aims to examine the effectiveness and implementation outcomes of integrating ePROMs into routine care for children with cancer. The study has been designed to investigate clinical, organisational and systems-level issues, and to develop strategies for each to ensure full integration and uptake of the RESPONSE system. Understanding these implementation outcomes are likely to yield meaningful information to inform future multi-site implementation and evaluation.

### Involvement of stakeholders

From conception, the project has used co-design principles [51] to develop the RESPONSE system. Our wide stakeholder engagement has included children and families frontline medical, nursing and allied health staff; as well as hospital administrators and executives. The RESPONSE project is governed by a clinical reference group consisting of senior oncology medical and nursing staff that meets at least four times per year to provide oversight regarding the implementation plan for the project. There are also formal mechanisms in place to obtain feedback from families through the ‘Oncology Family Forum’ which involves families of children currently undergoing cancer treatment. Through these processes, children and caregivers have endorsed the need for the RESPONSE system and subsequently the use of a mobile app to record information about their child’s cancer symptoms in a structured way.

### Limitations

There are inherent challenges with undertaking this type of research with children and caregivers. The heterogenous population which includes a wide range of developmental stages and ages, as well as diagnosis may limit planned analysis. The appropriate timing for delivery of SSPedi has not been established and this may effect the acceptability of the intervention. However to date, and despite interruptions due to COVID-19, > 50 % of recruited control participants have completed > 80 % of the measures. As a complex intervention, there may be additional limitations that effect the generalisability of both the intervention and the findings of this study.

## Data Availability

Not applicable.

## Abbreviations

CFIR: Consolidation Framework for Implementation Research
ePROM: Electronic Patient Reported Outcome Measure
ERIC: Expert Recommendation for Implementing Change
HREC: Human Research Ethics Committee
PROM: Patient Reported Outcome Measure
RE-AIM: Reach, Effectiveness, Adoption, Implementation, Maintenance
RESPONSE: Remote Symptom Management in Paediatric Oncology
SSPedi: Symptom Screening in Pediatrics
